# BCL6 attenuates renal inflammation via negative regulation of NLRP3 transcription

**DOI:** 10.1038/cddis.2017.567

**Published:** 2017-10-26

**Authors:** Dan Chen, Xiao-Qing Xiong, Ying-Hao Zang, Ying Tong, Bing Zhou, Qi Chen, Yue-Hua Li, Xing-Ya Gao, Yu-Ming Kang, Guo-Qing Zhu

**Affiliations:** 1 Key Laboratory of Cardiovascular Disease and Molecular Intervention, Department of Physiology, Nanjing Medical University, Nanjing, Jiangsu 211166, China; 2 Department of Pathophysiology, Nanjing Medical University, Nanjing, Jiangsu 211166, China; 3 Department of Physiology and Pathophysiology, Cardiovascular Research Center, Xi’an Jiaotong University School of Medicine, Xi’an 710061, China

## Abstract

Renal inflammation contributes to the pathogeneses of hypertension. This study was designed to determine whether B-cell lymphoma 6 (BCL6) attenuates renal NLRP3 inflammasome activation and inflammation and its underlying mechanism. Male spontaneously hypertensive rats (SHR) and Wistar-Kyoto rats (WKY) were used in the present study. Angiotensin (Ang) II or lipopolysaccharides (LPS) was used to induce inflammation in HK-2 cells, a human renal tubular epithelial (RTE) cell line. NLRP3 inflammasome was activated and BCL6 was downregulated in the kidneys of SHR. Either Ang II or LPS suppressed BCL6 expression in HK-2 cells. BCL6 overexpression in HK-2 cells attenuated Ang II-induced NLRP3 upregulation, inflammation and cell injury. The inhibitory effects of BCL6 overexpression on NLRP3 expression and inflammation were also observed in LPS-treated HK-2 cells. BCL6 inhibited the NLRP3 transcription via binding to the NLRP3 promoter. BCL6 knockdown with shRNA increased NLRP3 and mature IL-1*β* expression levels in both PBS- or Ang II-treated HK-2 cells but had no significant effects on ASC, pro-caspase-1 and pro-IL-1*β* expression levels. BCL6 overexpression caused by recombinant lentivirus expressing BCL6 reduced blood pressure in SHR. BCL6 overexpression prevented the upregulation of NLRP3 and mature IL-1*β* expression levels in the renal cortex of SHR. The results indicate that BCL6 attenuates Ang II- or LPS-induced inflammation in HK-2 cells via negative regulation of NLRP3 transcription. BCL6 overexpression in SHR reduced blood pressure, NLRP3 expression and inflammation in the renal cortex of SHR.

Hypertension is now recognized as a chronic, low-grade inflammatory disease in the kidneys and blood vessels.^1^ There is a correlation between the intensity of the renal inflammation and the severity of the blood pressure elevation in spontaneously hypertensive rats (SHR).^2^ Intraperitoneal administration of interleukin (IL)-10, an anti-inflammatory cytokine, normalized blood pressure in hypertensive pregnant rats.^3^ Melatonin reduces renal inflammation and attenuates hypertension in SHR.^4^ Resveratrol ameliorates renal injury by inhibiting renal inflammation in SHR.^5^ Angiotensin (Ang) II is a pro-inflammatory cytokine, which is one of the main mechanisms involved in hypertension-induced tissue damage.^6, 7^ Renal inflammation has an important role in the development of hypertension and may be a target for attenuating hypertension.^8, 9, 10^

Nucleotide-binding oligomerization domain-like receptor protein 3 (NLRP3) inflammasome is a multi-protein complex and is composed of NLRP3, apoptotic speck protein containing a caspase recruitment domain (ASC) and pro-caspase-1.^11^ It can be triggered by exogenous and endogenous stimuli, including pathogen-associated molecular patterns and damage-associated molecular patterns, both of which belong to pattern-recognition receptors (PRRs).^12^ Once stimulated, the NLRP3 inflammasome is assembled, and caspase-1 is activated, which processes pro-IL-1*β* into its mature form IL-1*β*, and thus triggers an inflammatory response.^11^ Under both physiological and pathological conditions, PRRs are widely expressed in the kidney.^13, 14^ NLRP3 inflammasome promotes renal inflammation and contributes to chronic kidney disease (CKD).^13^ NLRP3 deficiency ameliorated fructose-induced renal injury by reducing renal inflammation, fibrosis, albuminuria and hyperuricemia.^15^ Knockdown of LincRNA-Gm4419 attenuates NLRP3 inflammasome-mediated inflammation in diabetic nephropathy.^16^

B-cell lymphoma 6 (BCL6) functions as a sequence-specific transcriptional repressor and inhibits NF-*κ*B signaling by inhibiting binding of BCL6 to NF-*κ*B proteins and deregulating the expression levels of NF-*κ*B target genes, including IL.^17, 18^ BCL6-deficient mice develop severe and spontaneous T helper type 2 (Th2) inflammation and BCL6-deficient Treg cells are ineffective at controlling Th2 responses.^19^ BCL6 is considered as a therapeutic target for autoimmune diseases and cancer treatment.^20^ We hypothesized that BCL6 would inhibit renal inflammation via negative regulation of NLRP3 transcription and would be beneficial in attenuating hypertension. The present study was designed to determine whether BCL6 attenuates renal NLRP3 inflammasome activation and inflammation and its underlying mechanism.

## Results

### Renal NLRP3 inflammasome activation and BCL6 downregulation in SHR

**Figure 1:**
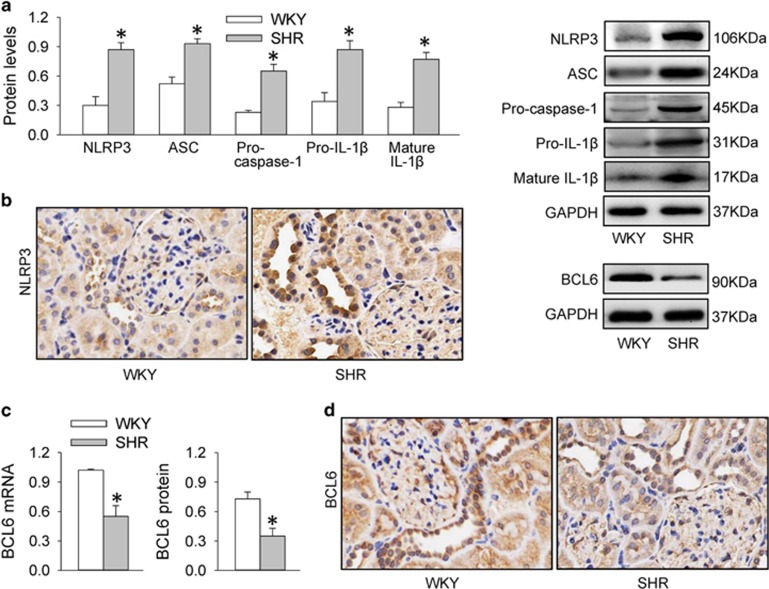
NLRP3 inflammasome activation and BCL6 expression in renal cortex of WKY and SHR. (**a**) Expression levels of NLRP3, ASC, pro-caspase-1, pro-IL-1*β* and mature IL-1*β* protein. (**b**) Immunohistochemistry for NLRP3 in the renal cortex. (**c**) Expression levels of Bcl6 mRNA and protein. (**d**) Immunohistochemistry for BCL6 in the renal cortex. Values are mean±S.E.M. *P<0.05 *versus* WKY. *n*=6

Expression levels of NLRP3, ASC and pro-caspase-1, the components of NLRP3 inflammasome, were upregulated in the kidneys of SHR compared with those of WKY. Pro-IL-1*β* and mature IL-1*β* protein contents in the kidneys were also increased in SHR (Figure 1a). Immunohistochemistry analysis in the renal cortex showed that NLRP3 expression in renal tubular epithelial (RTE) cells was increased in SHR (Figure 1b). These results indicate NLRP3 inflammasome activation and inflammation response in the kidneys of SHR. BCL6 mRNA and protein levels were downregulated in the kidneys of SHR compared with those of WKY (Figure 1c). Immunohistochemistry analysis in the renal cortex showed that BCL6 expression in RTE cells was reduced in SHR (Figure 1d). The results suggest a possibility that the downregulation of BCL6 may contribute to the NLRP3 inflammasome activation and inflammation response in the kidneys of SHR.

### Effects of BCL6 overexpression in Ang II-treated HK-2 cells

**Figure 2:**
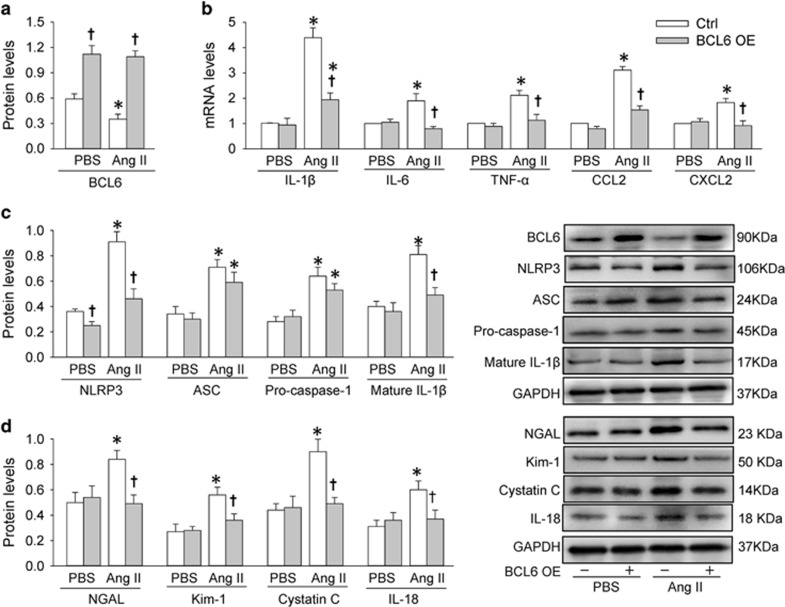
Effects of BCL6 overexpression (OE) on NLRP3 inflammasome activation in Ang II-treated HK-2 cells. The cells were treated with empty plasmid or BCL6 plasmid (1 *μ*g/ml) for 48 h followed by treatment with PBS or Ang II (1 *μ*M) for 12 h. (**a**) Expression of BCL6 protein. (**b**) IL-1*β*, IL-6, TNF-*α*, CCL2 and CXCL2 mRNA levels. (**c**) Expression levels of NLRP3, ASC, pro-caspase-1 and mature IL-1*β* protein. (**d**) Expression levels of NGAL, Kim-1, Cystatin C and IL-18 protein. Values are mean±S.E.M. *P<0.05 *versus* PBS; ^†^*P*<0.05 *versus* Ctrl. *n*=6

Ang II is a vaso-constrictive peptide that regulates blood pressure homeostasis and can cause inflammation in RTE cells.^6, 7^ In this study, Ang II was used to induce inflammation in HK-2 cells, a human RTE cell line, to mimic the renal inflammation in hypertension *in vitro*. Effects of BCL6 were examined after the HK-2 cells were treated with the BCL6 plasmid for 48 h to induce BCL6 overexpression, followed by treatment with Ang II for 12 h. Ang II reduced BCL6 expression in HK-2 cells. BCL6 plasmid significantly increased the BCL6 expression in both PBS- or Ang II-treated HK-2 cells, confirming the efficiency of the BCL6 overexpression protocol in the present study (Figure 2a). Ang II increased not only pro-inflammatory cytokines IL-1*β*, TNF-*α* and IL-6 mRNA levels but also chemokines CCL2 and CXCL2 mRNA levels, which were repressed by BCL6 overexpression in HK-2 cells (Figure 2b). The roles of BCL6 overexpression in attenuating inflammation were further confirmed by the facts that BCL6 overexpression reduced the NLRP3 and mature IL-1*β* protein expression levels in Ang II-treated HK-2 cells (Figure 2c). It is noted that BCL6 overexpression only reduced NLRP3 expression rather than the expression levels of other two components of NLRP3 inflammasome, ASC and pro-caspase-1 (Figure 2c), suggesting that the roles of BCL6 in preventing NLRP3 inflammasome activation and inflammation may depend on NLRP3 downregulation. NGAL, kim-1, cystatin C and IL-18 are generally used as markers of renal injury.^21^ Ang II increased NGAL, kim-1, cystatin C and IL-18 protein expression levels in HK-2 cells, which were prevented by BCL6 overexpression (Figure 2d), indicating that BCL6 attenuated Ang II-induced HK-2 cell injury. Ang II-induced phosphorylation of I*κ*B and its subsequent degradation were prevented by BCL6 overexpression (Supplementary Figure 1). Moreover, BCL6 overexpression inhibited Ang II-induced NF-*κ*B nuclear translocation in HK-2 cells (Supplementary Figure 2).

### Effects of BCL6 knockdown in Ang II-treated HK-2 cells

**Figure 3:**
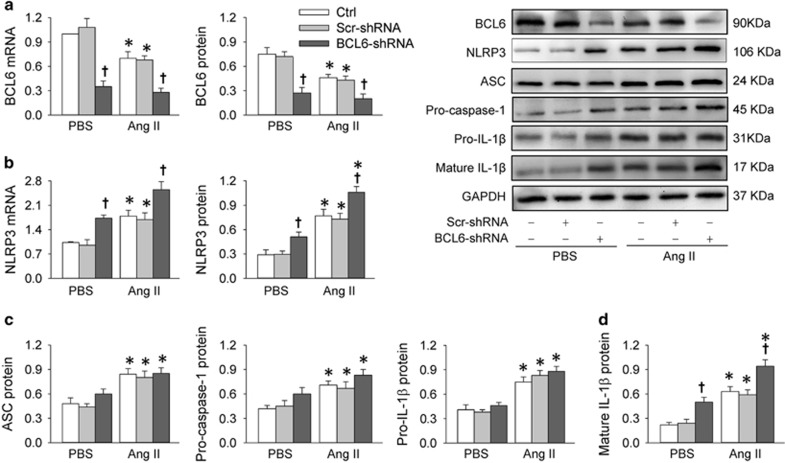
Effects of BCL6 knockdown on inflammasome activation in Ang II-treated HK-2 cells. The cells were treated with PBS (Ctrl), Scr-shRNA (scrambled shRNA) or BCL6-shRNA (1 *μ*g/ml) for 48 h followed by treatment with PBS or Ang II (1 *μ*M) for 12 h. (**a**) Expression levels of BCL6 mRNA and protein. (**b**) Expression levels of NLRP3 mRNA and protein. (**c**) Expression levels of ASC, pro-caspase-1 and pro-IL-1*β*. (**d**) Expression of mature IL-1*β* protein. Values are mean±S.E.M. *P<0.05 *versus* PBS; ^†^*P*<0.05 *versus* Ctrl or Scr-shRNA. *n*=6

Knockdown of BCL6 with short-hairpin RNA (shRNA) reduced the BCL6 mRNA and protein levels in both PBS- and Ang II-treated HK-2 cells, indicating the efficiency of the shRNA targeting BCL6 in the present study (Figure 3a). The BCL6 knockdown increased the NLRP3 expression at both protein and mRNA levels in PBS- or Ang II-treated HK-2 cells (Figure 3b) but had no significant effects on ASC, pro-caspase-1 and pro-IL-1*β* protein expression levels (Figure 3c). Furthermore, mature IL-1*β* protein was upregulated by the BCL6 knockdown in both PBS- and Ang II-treated HK-2 cells (Figure 3d).

### Effects of BCL6 overexpression in LPS-treated HK-2 cells

**Figure 4:**
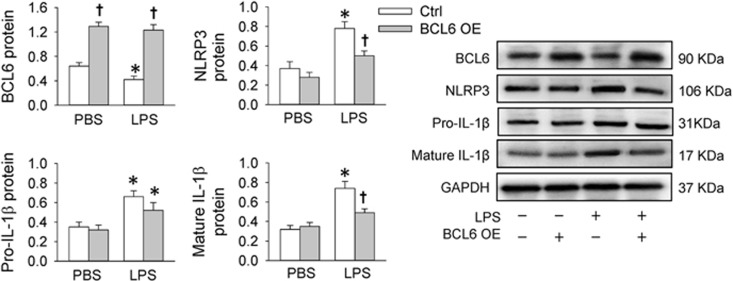
Effects of BCL6 overexpression (OE) on BCL6, NLRP3, pro-IL-1*β* and mature IL-1*β* expression levels in LPS-treated HK-2 cells. The cells were treated with empty plasmid or BCL6 plasmid (1 *μ*g/ml) for 48 h followed by treatment with PBS or LPS (5 *μ*g/ml) for 8 h. Values are mean±S.E.M. *P<0.05 *versus* PBS; ^†^*P*<0.05 *versus* Ctrl. *n*=6

An interesting question is whether BCL6 could inhibit the inflammation induced by other inflammatory agents. Lipopolysaccharide (LPS) is known to increase the release of inflammatory mediators, such as IL-1*β*.^22, 23, 24^ It has been found that LPS activates NLRP3 inflammasome in mouse lung vascular endothelial cells.^25^ Thus the effects of BCL6 on LPS-induced inflammation and NLRP3 expression were examined *in vitro*. LPS reduced BCL6 expression in HK-2 cells. BCL6 overexpression repressed the LPS-induced upregulation of NLRP3 and mature IL-1*β* rather than that of pro-IL-1*β* in HK-2 cells (Figure 4).

### BCL6 binds to NLRP3 promoter and inhibits NLRP3 transcription

**Figure 5:**
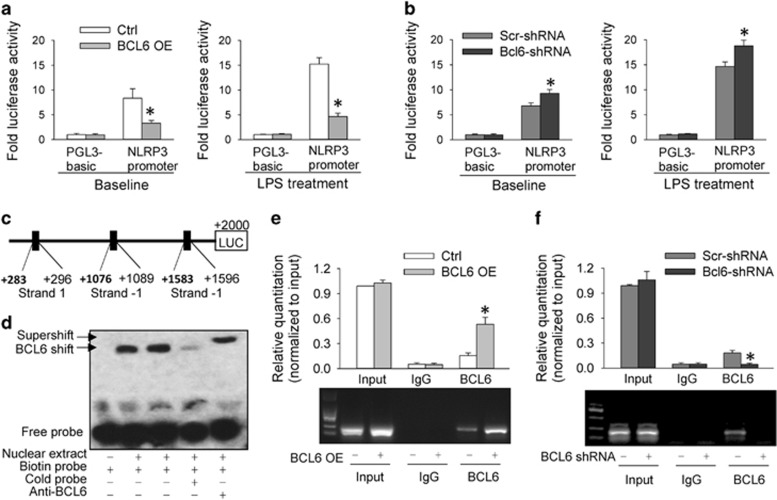
BCL6 binds to NLRP3 promoter and inhibits NLRP3 transcription. (**a**) Effects of BCL6 overexpression (1 *μ*g/ml) on luciferase activities in 293ET cells without or with LPS treatment (100 ng/ml). (**b**) Effects of BCL6-shRNA (1 *μ*g/ml) on luciferase activities in HK-2 cells without or with LPS treatment (100 ng/ml). (**c**) Potential BCL6-binding sites predicted by bioinformatics analysis (strand 1 stands for meaningful site, while strand −1 stands for meaningless site). (**d**) EMSA was performed with the biotin-labeled NLRP3 oligonucleotides containing BCL6-binding site (+283/+296) in HK-2 cells. (**e**) ChIP analysis showing the effects of BCL6 overexpression (1 *μ*g/ml) on the BCL6 binding to the +283/+296 site of NLRP3 promoter region in HK-2 cells. (**f**) ChIP analysis showing the effects of BCL6-shRNA (1 *μ*g/ml) on the BCL6 binding to the +283/+296 site of NLRP3 promoter region in HK-2 cells. Values are mean±S.E.M. *P<0.05 *versus* Ctrl or Scr-shRNA. *n*=6

Based on the finding that BCL6 represses targeted genes by binding the response element in the promoter region,^26^ we further investigated whether BCL6 could inhibit the NLRP3 expression at the transcription level. Dual luciferase report assay showed that BCL6 overexpression in 293ET cells inhibited NLRP3 transcription in both baseline state and LPS-treated state (Figure 5a and Supplementary Figure 3), while BCL6 knockdown in HK-2 cells promoted NLRP3 transcription in both baseline state and LPS-treated state (Figure 5b). Bioinformatics analysis (http://jaspar.binf.ku.dk/) predicted three potential BCL6 binding sites (+283/+296, +1076/+1089 and +1583/+1596) in human NLRP3 promoter. Two binding sites (+1076/+1089, +1583/+1596) were meaningless because their strands were −1 (Figure 5c). So we focused on the potential BCL6 binding site (+283/+296) in the promoter region. Electrophoretic mobility shift assay (EMSA) was used to analyze BCL6-binding site (+283/+296) in the nuclear extract from HK-2 cells (Figure 5d). BCL6 protein was bound to the labeled probe (lanes 2 and 3), and the binding was reduced with the unlabeled probe competition (lane 4). Importantly, incubation with BCL6 antibody supershifted the protein-DNA complex (lane 5), indicating the specificity of BCL6 binding. The results indicate that BCL6 binding to the promoter regions of NLRP3 gene in HK-2 cells were at position +283/+296 of the transcription start site. Chromatin immunoprecipitation (ChIP) assays were performed to confirm whether BCL6 occupies the promoter site at +283 / +296 *in vivo*. It confirmed the enrichment of BCL6 on NLRP3 promoter where BCL6 exerted its effect following BCL6 overexpression (Figure 5d). BCL6 knockdown reduced the occupation of BCL6 at the promoter site of NLRP3 (Figure 5e). These results indicate that BCL6 binds to the NLRP3 promoter and negatively regulates the NLRP3 expression.

### Effects of BCL6 overexpression in WKY and SHR

**Figure 6:**
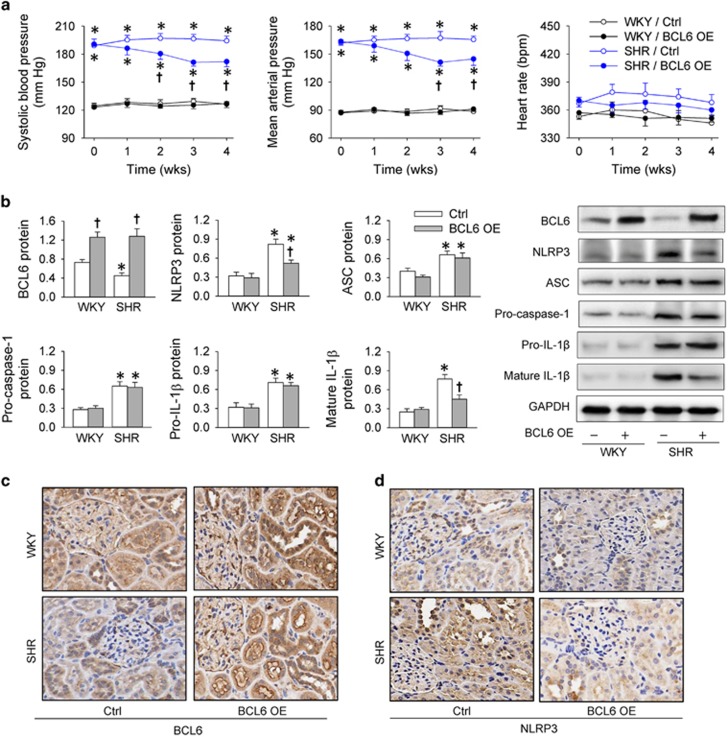
Effects of BCL6 overexpression (BCL6 OE) in WKY and SHR. (**a**) Systolic blood pressure, mean arterial pressure and heart rate. (**b**) Expression levels of BCL6, NLRP3, ASC, pro-caspase-1, pro-IL-1*β* and mature IL-1*β* in the renal cortex. (**c**) Immunohistochemistry for BCL6 in the renal cortex. (**d**) Immunohistochemistry for NLRP3 in the renal cortex. Values are mean±S.E.M. *P<0.05 *versus* WKY; ^†^*P*<0.05 *versus* Ctrl. *n*=6

BCL6 overexpression was induced by recombinant lentivirus-expressing BCL6 in WKY and SHR. The BCL6 overexpression had no significant effects on systolic blood pressure (SBP) and mean arterial pressure (MAP) in WKY but caused a persistent reduction in SBP (−19.5±4.1 mm Hg) and MAP (−23.3±3.9 mm Hg) in SHR, reaching their maximal effects at about 3 weeks (Figure 6a). The efficiency of BCL6 overexpression was confirmed by the upregulation of BCL6 expression in the renal cortex of both WKY and SHR (Figure 6b). BCL6 overexpression prevented the upregulation of NLRP3 and mature IL-1*β* expression levels but had no significant effects on the upregulation of ASC, pro-caspase-1 and pro-IL-1*β* expression levels in the renal cortex of WKY and SHR (Figure 6b). Immunohistochemistry analysis in the renal cortex showed that recombinant lentivirus-expressing BCL6 effectively increased the BCL6 expression, primarily in RTE cells, in both WKY and SHR (Figure 6c), and that the upregulation of NLRP3 expression in the renal cortex of SHR was prevented by the BCL6 overexpression (Figure 6d). Serum creatinine (SCr) level was increased in SHR, which was reduced by BCL6 overexpression (Table 1).

## Discussion

Renal inflammation has an important role in the development of hypertension.^8, 9, 10^ BCL6 gene encodes a protein containing six C-terminal zinc-finger motifs and an N-terminal POZ domain, and the BCL6 protein acts as a sequence-specific transcriptional repressor.^26^ BCL6 negatively regulates NF-*κ*B expression and thereby inhibits NF-*κ*B-mediated inflammation.^17^ NLRP3 inflammasome activation is involved in several inflammatory diseases.^27, 28^ However, it is unknown whether BCL6 would contribute to the NLRP3-mediated inflammation. The major novel findings in the present study are that BCL6 binds to the promoter region of NLRP3 and negatively regulates NLRP3 expression, BCL6 overexpression inhibits Ang II- or LPS-induced NLRP3 upregulation and inflammation and BCL6 knockdown exacerbates Ang II-induced NLRP3 upregulation and inflammation in HK-2 cells. More importantly, BCL6 expression in the renal cortex is downregulated in SHR, and BCL6 overexpression in SHR reduces blood pressure, inhibits the NLRP3 upregulation and inflammation in renal cortex of SHR.

BCL6 downregulation and NLRP3 inflammasome activation were found in the renal cortex of SHR, suggesting a possibility that BCL6 might inhibit the NLRP3 inflammasome activation. To test the hypothesis, effects of BCL6 on NLRP3 inflammasome and inflammation were examined *in vitro* and *in vivo*. Ang II has a crucial role in the pathogenesis of hypertension and related end-organ damage^29, 30^ and is involved in the pathogenesis of renal disease.^31^ Ang II-induced hypertension is a commonly used animal model of hypertension.^32, 33, 34^ It has been found that Ang II induces hypertensive renal inflammation in rat RTE cells.^6^ Thus Ang II-induced inflammation model in HK-2 cells was used to examine the effects of BCL6 *in vitro*. We found that BCL6 overexpression in HK-2 cells attenuated Ang II-induced inflammation evidenced by the reduction in the expression levels of pro-inflammatory cytokines (IL-1*β*, TNF-*α*, IL-6) as well as chemokines (CCL2 and CXCL2). Interestingly, BCL6 overexpression only prevented the Ang II-induced upregulation of NLRP3 but had no significant effects on the upregulation of other components of NLRP3 inflammasome (ASC and pro-caspase-1). These findings indicate that BCL6 attenuates Ang II-induced NLRP3 inflammasome activation and inflammation via reducing NLRP3 expression in HK-2 cells. The effects of BCL6 on NLRP3 expression was confirmed by that findings of dual luciferase activity assay, EMSA and ChIP, which indicate BCL6 binds to the NLRP3 promoter and negatively regulates NLRP3 expression. On the other hand, renal inflammation is known to be a crucial factor for renal injury.^10^ Inhibiting renal inflammation attenuates renal injury in SHR.^5^ There seems every reason to believe that the protective role of BCL6 in attenuating the Ang II-induced HK-2 cell injury may at least partially attribute to the attenuation of NLRP3 inflammasome activation and inflammation. It is noted that BCL6 also repressed the LPS-induced upregulation of NLRP3 and mature IL-1*β* in HK-2 cells, suggesting that the anti-inflammation effect of BCL6 is not limited to the Ang II-induced inflammation.

NF*κ*B, an inflammatory transcription factor, has an important role in the pathogenesis of hypertensive nephropathy.^35, 36^ NF*κ*B activation is triggered by I*κ*B phosphorylation and subsequent degradation, which causes NF*κ*B translocation to the nucleus and subsequent transcription of several target genes.^37^ The activation of NF*κ*B is closely linked to the physiological immunity and pathological inflammation.^38^ BCL6 is known to repress NF*κ*B activity in diffuse large B-cell lymphomas.^39^ NF*κ*B can directly binds to the promoter DNA sequences of NLRP3 and regulate NLRP3 expression in murine macrophages.^40^ NLRP3 inflammasome expression is driven by NF*κ*B in hepatocytes.^41^ Various stimuli that activate NF*κ*B can induce the assembly of NLRP3 inflammasome and then generate IL-1*β* secretion.^28^ In the present study, Ang II reduced BCL6 expression and increased I*κ*B*α* phosphorylation and p65-NF*κ*B nuclear translocation, which were prevented by BCL6 overexpression in HK-2 cells. These results indicate that BCL6 reduces p65 nuclear translocation and subsequent NLRP3 expression.

Knockdown of BCL6 increased NLRP3 and mature IL-1*β* expression levels but had no significant effects on ASC, pro-caspase-1 and pro-IL-1*β* in both PBS- and Ang II-treated HK-2 cells. The findings indicate that endogenous BCL6 is an inhibitor of NLRP3 inflammasome activation and inflammation via inhibiting the NLRP3 transcription in both physiological state and Ang II-induced inflammatory state. Thus the reduced BCL6 expression in the renal cortex of SHR may contribute to the renal inflammation in SHR. To confirm the important roles of BCL6 in NLRP3 inflammasome activation and inflammation in the renal cortex of SHR, we examined the therapeutical effects of BCL6 overexpression *in vivo*. Just as expected, BCL6 overexpression attenuated NLRP3 expression and inflammation in the renal cortex of SHR. More interestingly, BCL6 overexpression caused a slow and persistent reduction in blood pressure in SHR. The anti-inflammation effect of BCL6 in the renal cortex may partially attribute to its slow depressor effect in SHR. It is known that chronic vascular inflammation is involved in the pathogenesis of hypertension.^42, 43^ The effect of BCL6 in vascular inflammation needs further investigation in hypertension.

In summary, BCL6 inhibits NLRP3 expression and inflammation in Ang II- or LPS-treated human RTE cells via inhibiting NLRP3 transcription. BCL6 overexpression attenuates hypertension and inflammation in the renal cortex of SHR. BCL6 may be a potential target in the intervention of hypertensive renal inflammation.

## Methods

### Animals

Male SHR and WKY aged 13 weeks were obtained from Vital River Laboratory Animal Technology Co. Ltd (Beijing, China). Experiments were approved by the Experimental Animal Care and Use Committee of Nanjing Medical University, and the Guide for the Care and Use of Laboratory Animal published by the US National Institutes of Health (NIH publication, Eighth edition, 2011). The rats were housed in a temperature-controlled room with a 12-h light/dark cycle and a free access to standard chow and tap water.

### HK-2 cell culture

HK-2 cells were purchased from American Type Culture Collection (Rockville, MD, USA). The cells were cultured in medium consisting of DMEM/F12 (Wisent Inc., Montreal, Canada) supplemented with 10% FBS and antibiotics (100 units/ml penicillin and 100 mg/ml streptomycin) under a condition at 37 °C in a humidified air containing 5% CO_2_. The cells were continuously passaged at intervals of 2–3 days.^44, 45^

### Inflammation models in HK-2 cells

Either Ang II or LPS is commonly used to induce inflammation *in vitro*. The concentration of Ang II or LPS was selected according to previous studies. In Ang II-induced inflammation model, HK-2 cells were treated with 1 *μ*M of Ang II or PBS for 12 h.^44, 46^ In LPS-induced inflammation model, HK-2 cells were treated with 5 *μ*g/ml of LPS or PBS for 8 h.^47, 48^

### BCL6 overexpression in HK-2 cells

For plasmid construction, the BCL6-gene cDNA cloned by PCR was inserted into CMV-MCS-T2A-EGFP vectors (Hanbio Biotechnology Co., Ltd., Shanghai, China) and the plasmid was verified by sequencing. HK-2 cells were seeded in the six-well plates at a density of 1 × 10^5^ cells/ml. After 80% confluent, the HK-2 cells were transfected with BCL6 overexpression plasmid or pcDNA3.1 plasmid (1 *μ*g/ml) using lipofectamine 3000 (Invitrogen, Carlsbad, CA, USA). The transfected HK-2 cells were grown in 5% CO_2_ incubator at 37 °C for 48 h before administration of Ang II or LPS to induce inflammation.

### Knockdown of BCL6 in HK-2 cells

BCL6 was knockdown with BCL6 shRNA in HK-2 cells. The sequence used to silence BCL6 was 5′-AGTGAAGCAGAGATGGTTT-3′. Scramble-shRNA was used as control (Ctrl) and its sequence was 5′-TTCTCCGAACGTGTCACGT-3′. (GeneChem, Shanghai, China) HK-2 cells were transfected with BCL6-shRNA or scramble-shRNA when grown to 80% confluence. The transfected HK-2 cells were grown in 5% CO_2_ incubator at 37 °C for 48 h before administration of Ang II to induce inflammation.

### BCL6 overexpression in WKY and SHR

BCL6 overexpression lentivirus vector and enhanced red fluorescent protein (ERFP) lentivirus vector were constructed by Obio Technology (Shanghai) Corp., Ltd. (Shanghai, China). The induction of the lentivirus was performed as previously reported.^49, 50^ Simply, WKY and SHR aged 13 weeks were anaesthetized with pentobarbital sodium (50 mg/kg, i.p.). The left kidney was exposed via flank incision in aseptic manipulation, and recombinant lentivirus-expressing BCL6 or ERFP (2 × 10^9^ TU/ml, 50 *μ*l) were injected into different sites on the dorsal part of the left renal cortex via a 30-G needle. The abdomen was then closed. After 2 weeks, intravenous injection of recombinant lentivirus-expressing BCL6 or ERFP (2 × 10^9^ TU/ml, 50 *μ*l) was carried out via tail vein to ensure the adequate upregulation of BCL6 in kidneys. Acute experiments were carried out 4 weeks after the lentivirus induction.

### EMSA analyses

EMSA experiments were performed as previously described.^51, 52^ Simply, HK-2 cells (1 × 10^5^) were seeded into six-well plates before nuclear protein extraction. Nuclear extracts were prepared using the Nuclear and Cytoplasmic Extraction Kit (KeyGEN BioTECH, Nanjing, China).The sequences of the oligonucleotides used were 5′-CCTTTTTCTGGAGAATGGGG-3′ (+283/+296), and the oligonucleotide probes were labeled with biotin. In competitive binding assays, unlabeled oligonucleotides were added at 100-fold molar excess. For supershift assay, 2 *μ*g anti-BCL6 antibody was added.

### Real-time PCR

Total RNA was separated with Trizol reagent (Life Technologies, Gaithersburg, MD, USA) according to the manufacturer’s instruction. Reverse transcriptase reactions were performed using the PrimeScript RT reagent Kits. RT-PCR was performed using Quantitative PCR with SYBR Premix Ex Taq TM (Takara, Otsu, Shiga, Japan) and ABI PRISM 7500 sequence detection PCR system (Applied Biosystems, Foster City, CA, USA).^53^ The expression of mRNA was calculated using the comparative cycle threshold (Ct) method where the relative quantization of target transcript levels was determined by subtracting Ct values of target genes from Ct values of GAPDH. The sequences of primers are listed in Supplementary Tables S1 and S2).

### Western blotting analysis

Samples were homogenized in lysis buffer, and the supernatant was extracted for measurement of total protein with a Protein Assay Kit (BCA; Pierce, Santa Cruz, CA, USA). Equal amounts of total protein were separated in SDS-PAGE and transferred to PVDF membranes in Trisglycine methanol buffer. The bands were visualized using the enhanced chemiluminescence. GADPH was developed as a loading control to normalize the data. The antibodies against NLRP3, ASC, pro-caspase-1, IL-1*β*, Kim-1 and IL-18 were purchased from Abcam (Cambridge, MA, USA). Antibody against BCL6 was obtained from Cell Signaling Technology (Beverly, MA, USA). Antibodies against NGAL and Cystatin C were purchased from Bioworld Technology, Ltd (Nanjing, Jiangsu, China). Antibody against GAPDH was obtained from Santa Cruz Biotechnology (Santa Cruz, CA, USA).

### Immunohistochemistry of the kidney in rats

Immunohistochemistry for kidney was carried out as previously reported.^54, 55^ Simply, the left kidney was cut in half transversely, fixed in 10% formalin and embedded in paraffin. The kidney sections (5 *μ*m) were deparaffinized, rehydrated, blocked with 3% BSA and incubated with anti-BCL6 antibody (GeneTex Inc., Irvine, CA, USA) or anti-NLRP3 antibody (Novus Biologicals, Littleton, CO, USA). Then biotinylated secondary antibodies were used and followed by 3,3’-diaminobenzidine (DAB) solution to detect the avidin–biotin complex signal. Counterstaining was then performed before examination under a light microscope (DP70, Olympus, Tokyo, Japan).

### Luciferase activity assay

293ET cells were cultured on 96-well plate in DMEM with FBS (10%), penicillin (100 units/ml) and streptomycin (100 mg/ml) at 37 °C in a 5% CO_2_ humidified incubator. Then the cells were co-transfected with 1 *μ*g/ml NLRP3 promoter plasmid or pGL3-basic vehicle vector, 1 *μ*g/ml BCL6 overexpression plasmid or pcDNA3.1 for 24 h. Luciferase activities were measured and normalized to that of Renilla luciferase.

### Chromatin immunoprecipitation

ChIP assay were performed as previously described.^56^ Simply, HK-2 cells were transfected with BCL6 overexpression plasmid or pcDNA3.1 for 48 h and then they were crosslinked with 1% paraformaldehyde for 10 min before stopped with 125 mM glycine. After that, the samples were washed, lysed with cold PBS/protease inhibitor and collected. Chromatin was sheared by 10 s of sonication interposed with 10 s pauses, repeated 15 times. Immunoprecipitation was performed overnight at 4 °C, via 5 *μ*g of anti-BCL6 antibodies or immunoglobulin G (Santa Cruz Biotechnology). The precipitated DNA fragments were amplified by PCR. The following primers were used for NLRP3 Chip PCR, forward: 5′-TCTCCATTGTGTCTTCTTGGTG-3′ reverse: 5′-CTGGGTGACAAGAGCAAGACT-3′.

### Measurement of serum blood urea nitrogen and creatinine

Blood samples were obtained from aorta. Serum blood urea nitrogen and SCr concentrations were examined with the Urea Assay Kits and Creatinine Assay Kit following the manufacturer’s instructions (Nanjing Jiancheng Bioengineering Institute, Nanjing, China).

### Blood pressure measurement

Blood pressure of tail artery was measured in conscious state using a noninvasive computerized tail-cuff system (ADInstruments, Sydney, New South Wales, Australia) as previously reported.^57^ The average value of six measurements was calculated as the blood pressure of each rat.

### Statistical analysis

Comparisons between two groups were made by Student’s *t*-test. One-way or two-way ANOVA followed by *post hoc* Bonferroni test was used when multiple comparisons were made. All data were expressed as mean±S.E.M. A value of *P*<0.05 was considered statistically significant.

## Publisher’s Note

Springer Nature remains neutral with regard to jurisdictional claims in published maps and institutional affiliations.

## Figures and Tables

**Table 1:** Effects of BCL6 overexpression in WKY and SHR

	**WKY**		**SHR**	
	**Ctrl**	**BCL6 OE**	**Ctrl**	**BCL6 OE**
BW, g	354.3±7.6	349.1±7.2	323.8±6.8*	329.2±6.9*
HW, mg	1003±39	973±29	1142±25*	1054±28*^†^
KW, mg	2475±81	2499±83	2379±79	2373±92
HW/BW, mg/g	2.83±0.08	2.79±0.06	3.54±0.12*	3.21±0.08*^†^
KW/BW, mg/g	6.98±0.14	7.16±0.21	7.36±0.27	7.2±0.18
Serum BUN, mM	5.99±0.48	5.47±0.48	11.98±1.12*	10.16±1.26*
Serum Cr, *μ*M	77.8±5.4	87.4±4.1	140.5±10.3*	110.3±8.3*^†^

BW, body weight; BUN, blood urea nitrogen; Cr, creatinine; KW, kidney weight. Values are mean±S.E.M. *P<0.05 *versus* WKY; ^†^*P*<0.05 *versus* Ctrl. *n*=6 for each group.
