# Supplementary material for: BCL6 attenuates renal inflammation via negative regulation of NLRP3 transcription
Source: Cell Death Dis. 2017 Oct 26;8(10):e3156–. doi: 10.1038/cddis.2017.567 (PMC5680929; doi:10.1038/cddis.2017.567)
Supplement: Supplementary Material [file cddis2017567x1.docx]

**Supplementary Materials**

## (3 supplementary figures & 2 supplementary tables)

**BCL6 attenuates renal inflammation via negative regulation of NLRP3 transcription**

Dan CHEN^a^, Xiao-Qing Xiong^a^, Ying-Hao ZANG^a^, Ying TONG^a^, Bing Zhou ^a^, Qi CHEN^b^, Yue-Hua LI^b^, Xing-Ya GAO^a^, Yu-Ming KANG^c^, Guo-Qing ZHU^a^*

## ^a^Key Laboratory of Cardiovascular Disease and Molecular Intervention, Department of Physiology, Nanjing Medical University, Nanjing, Jiangsu 211166, China; ^b^Department of Pathophysiology, Nanjing Medical University, Nanjing, Jiangsu 211166, China; ^c^Department of Physiology and Pathophysiology, Cardiovascular Research Center, Xi'an Jiaotong University School of Medicine, Xi'an 710061, China

**
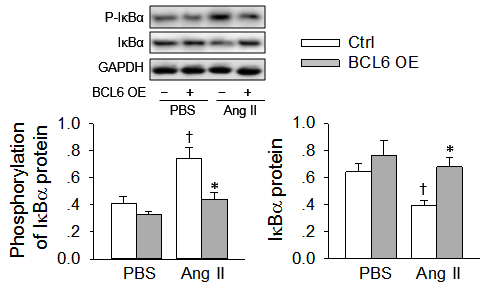
**

**Supplementary Figure 1** Eﬀects of BCL6 overexpression (OE) on Ang II‑induced IκBα phosphorylation and IκBα protein downregulation in HK-2 cells. The cells were treated with empty plasmid or BCL6 plasmid (1 μg/mL) for 48 h followed by treatment with PBS or Ang II (1 μM) for 12 h. Values are mean±SE. *P<0.05 vs. Ctrl. †P<0.05 vs. PBS. n=3.

**
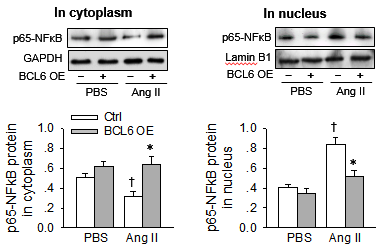
**

**Supplementary Figure 2** Eﬀects of BCL6 overexpression (OE) on Ang II‑induced p65 nuclear translocation in HK-2 cells. The cells were treated with empty plasmid or BCL6 plasmid (1 μg/mL) for 48 h followed by treatment with PBS or Ang II (1 μM) for 12 h. Values are mean±SE. *P<0.05 vs. Ctrl. †P<0.05 vs. PBS. n=3.

**
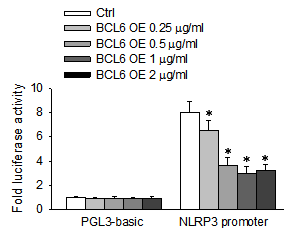
**

**Supplementary Figure 3** Dose effects of BCL6 overexpression on luciferase activities in 293ET cells. Values are mean±SE. *P<0.05 vs. Ctrl. n=3.

**Supplementary table 1** Primers for RT-PCR analysis in rats

|  | Primer | Sequence |
| --- | --- | --- |
| GAPDH | Forward | TGAGGCCGGTGCTGAGTATGT |
|  | Reverse | CAGTCTTCTGGGTGGCAGTGAT |
| BCL6 | Forward | TAGTGATGTTCTTCTCAACC |
|  | Reverse | TGGTCAGTGAAGATGCTGTAG |

**Supplementary table 2** Primers for RT-PCR analysis in human HK-2 cells

|  | Primer | Sequence |
| --- | --- | --- |
| GAPDH | Forward | AACAGCGACACCCACTCCTC |
|  | Reverse | GGAGGGGAGATTCAGTGTG |
| BCL6 | Forward | CGATGAGGAGTTTCGGGATGTC |
|  | Reverse | TTTCTGGGGGCTCTGTGGACTAAC |
| NLRP3 | Forward | TGATGATATGAGCAAAAGTAAAC |
|  | Reverse | CCCTCAGTTAGAGGATGTTC |
| IL-1β | Forward | ACGATGCACCTGTACGATCA |
|  | Reverse | TCTTTCAACACGCAGGACAG |
| IL-6 | Forward | CCTTCCAAAGATGGCTGAAA |
|  | Reverse | AGCTCTGGCTTGTTCCTCAC |
| TNF-α | Forward | AACCTCCTCTCTGCCATCAA |
|  | Reverse | CCAAAGTAGACCTGCCCAGA |
| CCL2 | Forward | TCTGTGCCTGCTGCTCATAG |
|  | Reverse | TGGAATCCTGAACCCACTTC |
| CXCL2 | Forward | CTCAAGAATGGGCAGAAAGC |
|  | Reverse | AAACACATTAGGCGCAATCC |
